# Pay It Forward: High School Video-based Instruction Can Disseminate CPR Knowledge in Priority Neighborhoods

**DOI:** 10.5811/westjem.2017.10.35108

**Published:** 2018-02-20

**Authors:** Marina Del Rios, Josiah Han, Alejandra Cano, Victor Ramirez, Gabriel Morales, Teri L. Campbell, Terry Vanden Hoek

**Affiliations:** * University of Illinois at Chicago – College of Medicine, Department of Emergency Medicine, Chicago, Illinois; † University of Washington, Department of Emergency Medicine, Seattle, Washington; ‡ University of Chicago Aeromedical Network, Chicago, Illinois

## Abstract

**Introduction:**

The implementation of creative new strategies to increase layperson cardiopulmonary resuscitation (CPR) and defibrillation may improve resuscitation in priority populations. As more communities implement laws requiring CPR training in high schools, there is potential for a multiplier effect and reach into priority communities with low bystander-CPR rates.

**Methods:**

We investigated the feasibility, knowledge acquisition, and dissemination of a high school-centered, CPR video self-instruction program with a “pay-it-forward” component in a low-income, urban, predominantly Black neighborhood in Chicago, Illinois with historically low bystander-CPR rates. Ninth and tenth graders followed a video self-instruction kit in a classroom setting to learn CPR. As homework, students were required to use the training kit to “pay it forward” and teach CPR to their friends and family. We administered pre- and post-intervention knowledge surveys to measure knowledge acquisition among classroom and “pay-it-forward” participants.

**Results:**

Seventy-one classroom participants trained 347 of their friends and family, for an average of 4.9 additional persons trained per kit. Classroom CPR knowledge survey scores increased from 58% to 93% (p < 0.0001). The pay-it-forward cohort saw an increase from 58% to 82% (p < 0.0001).

**Conclusion:**

A high school-centered, CPR educational intervention with a “pay-it-forward” component can disseminate CPR knowledge beyond the classroom. Because schools are centrally-organized settings to which all children and their families have access, school-based interventions allow for a broad reach that encompasses all segments of the population and have potential to decrease disparities in bystander CPR provision.

## INTRODUCTION

Each year 395,000 people suffer an out-of-hospital cardiac arrest (OHCA) in the United States.^1^ Shortening the time between OHCA onset and the first three links in the chain of survival—early access to emergency medical services (EMS), early cardiopulmonary resuscitation (CPR), and early defibrillation— is critical to improve survival outcomes.^2^ Multiple studies have demonstrated that layperson CPR increases chance of survival by 2–3 fold.^3^,^4^,^5^,^6^ The importance of immediate response by the public has been highlighted by the Institute of Medicine (IOM) report “Strategies to Improve Cardiac Arrest Survival: A Time to Act” (2015).^7^ One of the key recommendations of the IOM report was a call to “foster a culture of action through public awareness and training” to reduce the risk of irreversible neurologic injury and functional disability.^7^

Wide disparities in bystander CPR rates and OHCA outcomes persist, with some communities reporting a five-fold difference in survival.^1^,^8^,^9^,^10^ Residents who live in neighborhoods that are primarily Black, Hispanic, or low-income are more likely to have an OHCA, less likely to receive bystander CPR, and are less likely to survive.^8^ ^11^ The implementation of creative new strategies to increase layperson CPR and defibrillation may improve resuscitation in priority populations.^12^,^13^ No single training approach is comprehensive enough to eliminate these disparities. Most communities will only improve survival through a multifaceted, community-wide approach that may include teaching hands-only CPR for bystanders,^14^,^15^ emphasis on brief educational videos^16^ and video self-instruction,^17^,^18^ mandatory school-based training,^19^ and dispatcher-assisted CPR.^20^,^21^

One particularly high-yield approach for high-risk communities is the implementation of mandatory CPR training in high schools.^22^ The American Heart Association (AHA), the World Health Organization, and the IOM along with multiple other national and international advocacy groups have endorsed CPR training in high school as a key foundation to improve OHCA survival outcomes.^7^,^19^,^22^ The 2015 IOM report calls for state and local education departments to partner with training organizations and public advocacy groups to promote and facilitate CPR and automated external defibrillator (AED) training as a high school graduation requirement.^7^ Today communities across the U.S. have recognized the value of CPR training in high schools, and 36 states have enacted laws calling for mandatory training prior to graduation.^23^

The benefit of CPR training in high schools is understood as a long-term investment to ensure that multiple generations are trained and ready to act.^19^ However, a more immediate consequence of school-centered training may be the amplification of community CPR training and literacy as students become trainers for their household and circle of friends. 24,25 Students can be asked to “pay it forward” by sending them home with CPR training materials and assigning them the task of training friends and family members.

This pilot program sought to investigate the feasibility, knowledge acquisition, and dissemination of a high school-centered, CPR video self-instruction program with a “pay-it-forward” component in a low-income, urban, predominantly Black neighborhood with historically low bystander-CPR rates. Schools provide large-scale, centrally organized community settings accessible to both children and adult family members of all socioeconomic backgrounds. A student-mediated, CPR educational intervention may be an effective conduit to relay OHCA knowledge and preparedness in high-risk neighborhoods.

**Population Health Research Capsule**

What do we already know about this issue?

Victims of cardiac arrest from primarily Black, Hispanic, and low-income neighborhoods are less likely to receive bystander CPR, and are less likely to survive.

What was the research question?

Can a high school-centered, video-based CPR educational program disseminate CPR knowledge in priority neighborhoods?

What was the major finding of the study?

The program increased CPR literacy in students and amplified community literacy as students became community CPR trainers.

How does this improve population health?

Widespread adoption of high school, CPR-training programs can amplify CPR literacy and may improve bystander CPR rates and cardiac arrest outcomes in priority neighborhoods.

## METHODS

### Program Setting and Population

The neighborhood of West Garfield Park in Chicago, Illinois, had been previously identified by our group using spatial epidemiologic clustering techniques as a community with high rates of cardiac arrest and low rates of bystander CPR.^26^ The school selected for our CPR training intervention was Providence St. Mel, a Catholic high school located in the heart of West Garfield Park. Students enrolled in this school are 99.8% Black, and 61.8% come from low-income households. Participant enrollment was by purposeful convenience sample of ninth and tenth grade high school students in their physical education class period.

### Human Participant Protection

This study was determined exempt from review by the Office for the Protection of Research Subjects of the University of Illinois at Chicago.

### Program Design

The “pay it forward” CPR training program for high schools consisted of two parts: (1) a classroom-based, video-directed, learning intervention with an instructor-facilitated, practical skills module, and (2) a student-facilitated, in-home educational intervention using CPR Anytime Kits^TM^.

#### Classroom intervention: Instructor-facilitated, video self-training

Two in-class training sessions of 45 minutes each during physical education period were delivered by two volunteer trainers with AHA Basic Life Support Certification and at least 30 hours of experience teaching Hands-Only CPR. During the first 10 minutes, students completed a written multiple-choice CPR and AED knowledge survey (Appendix A). Survey questions were adapted from a previously validated survey instrument used by the Denver High Arrest Neighborhoods to Decrease Disparities in Survival (HANDS) Program.^27^ Upon completion the pre-intervention survey was collected from the class, and each student received an AHA CPR Anytime^TM^ video self-instruction kit. This previously validated kit includes an instructional DVD (in English and Spanish) and inflatable mannequins with a built-in feedback mechanism that clicks with adequate compression depth.^28^ The next 20–30 minutes consisted of instructor-facilitated, video-based instruction. The CPR Anytime^TM^ kit DVD was shown in front of the class while the instructors were on hand to answer questions and supervise hand positioning. Students were also taught how to operate an AED and practiced using a trainer AED. During the last 5–10 minutes of the class period, students completed a knowledge assessment survey, which was a replica of the pre-training survey. To protect participants’ privacy, pre- and post-training surveys did not include personal identifiable information.

#### “Pay it forward”

As homework, students were required to teach at least three friends or family members by using the video self-instruction kits in a train-the-trainer model. Students were asked to replicate their classroom experience by first administering pre surveys, followed by showing the self-instruction video and coaching participants through the practical portion at home, and finally administering the post survey to participants. Students were to return their data collection form and family and friends pre-/post-test surveys at two weeks to receive full credit. The surveys completed by family and friends did not include personal, identifiable information.

### Measurements and Outcomes

The primary outcome was knowledge gained by high school students trained in school as measured by improvement in the knowledge survey. The secondary outcome measure was dissemination into the neighborhood as measured by (1) the number of people trained per student, and (2) knowledge acquisition by friends and family members trained at home.

### Data Analysis

Descriptive statistics are presented as frequencies and percentages. We used Pearson’s chi-square analysis to determine whether differences in pre- vs. post-knowledge surveys were statistically significant. Analyses were performed with Stata version 14.2 (StataCorp LP, College Station, TX).

## RESULTS

Seventy-one students participated in the classroom-based educational intervention and took training kits home to teach friends and family. Sixty-nine completed the pre-training survey. All 71 students completed the post-training survey and took home the video self-instruction kits to “pay it forward.”

Because the surveys did not request personal identifiable information, we analyzed survey results in aggregate. Table 1 compares the percent of correct answers in the pre-training survey and post-training survey. The aggregate percent of correct answers increased from 58% pre training to 93% post training (p < 0.0001). An increase in correct responses was observed for several key concepts including adequate compression rate (from 20% to 96%, p < 0.0001), compression depth (from 25% to 92%, p < 0.0001), appropriate circumstances to perform COCPR (from 36% to 94%, p < 0.0001), and ease of defibrillator use (from 28% to 94%, p < 0.0001).

These 71 students in turn trained 347 friends and family members for a total of 418 people trained. On average, each student trained an additional 4.9 people (347/71) and each kit was used to train 5.9 in total (418/71). Pre-training surveys were completed by all 347 “pay-it-forward” participants; 344 also completed the post-training survey.

Table 2 summarizes knowledge acquisition for the “pay-it-forward” arm of the study. There was a statistically significant increase in the aggregate number of correctly answered questions between pre- and post-training surveys (58% to 82%, p < 0.0001). An increase in correct responses was observed for key concepts including compression rate (32% to 66%, p < 0.0001), compression depth (37% to 78%, p < 0.0001), when is it appropriate to use COCPR (46% to 74%, p < 0.0001) and ease of using a defibrillator (28% to 98%, p < 0.0001).

## DISCUSSION

To our knowledge, this is the first study conducted in the U.S. to demonstrate that a high school-centered, CPR educational intervention with a “pay-it-forward” component can disseminate CPR knowledge beyond the classroom and reach into low-income, minority neighborhoods. High school participants and subsequently trained friends and family demonstrated a statistically significant improvement in aggregate scores. Moreover, students trained an average of 4.9 additional people, demonstrating the potential for a multiplier effect.

In a study from Denmark, mass distribution of similar video self-instruction kits resulted in dissemination to an average of 2.5 additional people per student (only 19.8% of participants responded to questionnaires on whom they trained). 24 In another study from Norway with a better survey response rate of 78%, an additional 2.8 people were trained per student participant.^25^ Students in our training intervention outperformed their Denmark and Norway counterparts. Our survey response rate was 97% (69/71), and students taught on average an additional 4.9 people. Moreover, all participants, students and family and friends, demonstrated significant CPR and AED knowledge increase compared to baseline.

Opponents to compulsory training cite cost and time as barriers to implementation.^29^,^30^ However, an investment of one 45- to 60-minute period every school year is sufficient to ensure widespread CPR knowledge.^31^,^32^ In our study, training was completed in a 45-minute physical education class period, with minimum loss of standard curriculum time, and at low cost. With a retail price of $38.50,^33^ the estimated cost per person trained in our pilot program was $6.54. By using video-based learning with an inflatable mannequin, schools can teach Hands-Only CPR skills in a single class period at low cost and with good knowledge acquisition.

Financially restricted schools and communities may not be able to invest in individual training kits for each high school student to take home or even for use in school. A more cost-effective model may include video-based training with use of shared CPR mannequins in the classroom setting. Instead of taking kits home for skills training, students can pay it forward and instruct others by using video and web-based learning platforms without skills practice. Previous research in Arizona has demonstrated that bystanders who learned CPR by watching a 60-second video without skills practice had significantly improved responsiveness, chest compression rate, and decreased hands-off intervals compared to no training.^16^

The “pay-it-forward” model also provides an opportunity for high school students to reinforce their knowledge of the chain of survival. Medical students in Germany demonstrated that their own CPR skills improved by teaching schoolchildren.^34^ Another study from Belgium demonstrated that instructing schoolchildren to teach Basic Life Support (BLS) to their relatives and friends led to a more positive attitude of the adults towards bystander CPR.^35^ A CPR educational intervention in which high school students become teachers to friends and family can reinforce student knowledge while empowering youth to become community health advocates. As of the drafting of this study, 36 states including Illinois have made CPR a mandatory component of the public high school curriculum.^23^ The widespread adoption of CPR training in schools represents a long-term investment to ensure that multiple generations are trained and ready to act.^19^ An immediate benefit is the potential impact of adolescents as lay rescuers. 19 Another short-term benefit not well investigated is the potential for an immediate multiplier effect by reaching out of the classroom and into the communities served by the schools.

One successful example of health information flowing from child to parent is the Hip Hop for Stroke (HHS) program, a school-based, multimedia, stroke-literacy intervention targeting children aged 8–12 in Central Harlem.^36^ HHS improved knowledge of stroke symptoms and intent to activate 9-1-1 in children participants while increasing parental stroke literacy.^37^ While the concept of child-mediated health education is not new, its application to OHCA remains novel and untested as a major strategy to address significant disparities in outcome by community. Because schools provide large-scale, centrally organized settings accessed by people from all ranges of the social spectrum, a high school-centered, communitywide CPR training program has remarkable potential for reach into communities that would otherwise be hard to reach by traditional CPR education efforts.

There is significant evidence regarding the high efficacy of child-mediated CPR education. Previous survey studies of witnesses to OCHA have demonstrated that any previous CPR training is a predictor of CPR performance.^38^ ^39^ Moreover, parallel efforts in faith-based, community-based, and employment organizations to teach Hands-Only CPR and share that knowledge with their constituents may have a ripple effect in communities with low bystander-CPR rates.^40^ ^41^ ^42^

Multifaceted, community-based approaches aimed at strengthening the link in the chain of survival have been successful at increasing bystander-CPR rates and, subsequently, cardiac arrest survival.^43^,^44^,^45^ To eliminate disparities in bystander CPR provision, public education campaigns must prioritize neighborhoods with the highest need as identified using public health surveillance tools such as registries. 27, 46 The effect in communities found to have a high incidence of cardiac arrest and little-to-no incidence of bystander CPR could be exponential.

## LIMITATIONS

A significant limitation of this study was the inability to determine individual knowledge acquisition given that surveys did not include personal, identifiable information. However, the marked and statistically significant improvement in aggregate scores suggest that a video self-instruction, CPR-training program with a “pay-it-forward” component can increase understanding of the indications for and the steps to perform CPR.

Another limitation was the inability to ensure quality control of the pay-it-forward component. It is uncertain whether students provided the answers to the people that they trained or if the increase in the post-intervention scores truly reflected knowledge increase. It is also unclear whether knowledge will translate into adequate technique or increased bystander CPR and AED use. Despite these limitations, our “pay-it-forward” model is an inexpensive, novel strategy to disseminate CPR and AED knowledge in priority neighborhoods with limited access to traditional CPR training courses.

## CONCLUSION

Our student-led, pay-it-forward model using video self-instruction kits is an efficient training intervention to deliver bystander CPR and AED educational intervention in low-income, minority neighborhoods. Because schools are centrally organized settings to which all children and their families have access, school-based interventions allow for a broad reach that encompasses all segments of the population and have potential to decrease disparities in provision of bystander CPR and use of AEDs. Future research will seek to determine long-term knowledge retention of this educational intervention, as well as measure associated trends in bystander CPR within communities reached.

## Supplementary Information



## Figures and Tables

**Table 1:** Pre- and post-training survey data demonstrating statistically significant increase in knowledge acquisition of cardiopulmonary resuscitation (CPR) for students trained in high school.

Question	Before CPR training N =69		After CPR training N = 71		P value
	
	Correct	Percent	Correct	Percent	
It is better to do any CPR than to do no CPR?	58	84%	71	100%	0.0005
How do you check a person for a response?	49	71%	61	86%	0.0317
It is appropriate to use Hands-Only CPR in which situation?	25	36%	67	94%	< 0.0001
When providing Hands-Only CPR one should push on the victim's:	68	99%	69	97%	0.5764
What are the correct steps for providing Hands-Only CPR?	50	72%	55	77%	0.4945
When using an automated external defibrillator (AED) one should:	55	80%	71	100%	0.0001
How fast should you compress when doing Hands-Only CPR?	14	20%	68	96%	<0.0001
What does an automated external defibrillator (AED) do?	45	65%	68	96%	<0.0001
How deep should you do chest compressions when performing Hands-Only CPR?	17	25%	65	92%	<0.0001
How easy is it to use an automated external defibrillator (AED)?	19	28%	67	94%	<0.0001
Aggregate correct answers	400/690	58%	662/710	93%	<0.0001

**Table 2:** Pre- and post-training survey data demonstrating statistically significant increase in knowledge acquisition for friends and family members trained in cardiopulmonary resuscitation at home.

Question	Before CPR training N =347		After CPR training N = 344		P value
	
	Correct	Percent	Correct	Percent	
It is better to do any CPR than to do no CPR?	285	82%	321	93%	<0.0001
How do you check a person for a response?	192	55%	261	76%	<0.0001
It is appropriate to use Hands-Only CPR in which situation?	161	46%	256	74%	<0.0001
When providing Hands-Only CPR one should push on the victim's:	293	84%	316	92%	0.0026
What are the correct steps for providing Hands-Only CPR?	202	58%	267	78%	<0.0001
When using an automated external defibrillator (AED) one should:	271	78%	321	93%	<0.0001
How fast should you compress when doing Hands-Only CPR?	111	32%	228	66%	<0.0001
What does an automated external defibrillator (AED) do?	227	65%	289	84%	<0.0001
How deep should you do chest compressions when performing Hands-Only CPR?	129	37%	267	78%	<0.0001
How easy is it to use an automated external defibrillator (AED)?	152	44%	295	86%	<0.0001
Aggregate correct	2023/3470	58%	2821/3440	82%	<0.0001
