# Supplementary material for: Pay It Forward: High School Video-based Instruction Can Disseminate CPR Knowledge in Priority Neighborhoods
Source: West J Emerg Med. 2018 Feb 20;19(2):423–9. doi: 10.5811/westjem.2017.10.35108 (PMC5851521; doi:10.5811/westjem.2017.10.35108)
Supplement: Supplementary file 1 [file wjem-19-423-s001.pdf]

CPR Anytime Pre

1. What grade are you in?

2. What is your zip code?

3. It is better to do any CPR than to do no CPR?

- [ ] True
- [ ] False

4. How do you check a person for a response?

- [ ] Tap or shake a shoulder
- [ ] Shout or speak loudly and ask if they're okay
- [ ] Both a and b

5. It is appropriate to use Hands-Only CPR in which situation?

- [ ] A drowning victim
- [ ] An unconscious child
- [ ] A teen or adult in cardiac arrest

6. When providing Hands-Only CPR one should push on the victim's:

- [ ] Leg
- [ ] Mouth
- [ ] Center of the Chest

7. What are the correct steps for providing Hands-Only CPR?

- [ ] First dial 911 and then push hard and fast in the center of the victim's chest
- [ ] Push hard and fast in the center of the victim's chest then dial 911
- [ ] Give two breaths then dial 911

8. When using an automated external defibrillator (AED) you should:

- [ ] Apply pads to the victim's bare chest
- [ ] Apply pads over the victim's clothes
- [ ] Apply pads to the victim's arm and leg

9. How fast should you compress when performing Hands-Only CPR?

- [ ] Slowly, 40 times per minute
- [ ] Moderately, 75 times per minute
- [ ] Very fast, 100 times per minute
- [ ] Super fast, 125 times per minute
- [ ] Extremely fast, 200 times per minute

10. What does an automated external defibrillator (AED) do?

- [ ] Delivers an electric shock to restart the heart
- [ ] Does chest compressions
- [ ] Does mouth-to-mouth breathing
- [ ] Functions as a backup power generator
- [ ] Serves as a smoke and toxin detector

11. How deep should you do chest compressions when performing Hands-OnlyTM CPR?

- [ ] 1/2 inch
- [ ] 1 inch
- [ ] 2 inches
- [ ] 3 inches

12. How easy is it to use an automated external defibrillator (AED)?

- [ ] Easy- open the case and follow the voice prompts
- [ ] Medium- only those who have formal training should use it
- [ ] Hard- only medical professionals should use it
